# Aligned piezoelectric fibrous scaffolds for prevention of traumatic neuroma formation

**DOI:** 10.3389/fbioe.2025.1662072

**Published:** 2026-01-07

**Authors:** Yujie Chen, Xinyi Gu, Huiran Zang, Cai Wang, Guangxue Li

**Affiliations:** 1 Department of Plastic Surgery, Peking University People’s Hospital, Beijing, China; 2 Department of Plastic & Reconstructive Surgery, Center for Plastic & Reconstructive Surgery, Hangzhou Medical College, Zhejiang Provincial People’s Hospital (Affiliated People’s Hospital), Hangzhou, Zhejiang, China

**Keywords:** aligned fibrous scaffolds, piezoelectric effect, traumatic neuroma, electrospinning, Poly (L-lactide) (PLLA)

## Abstract

**Purpose:**

Traumatic neuroma, a painful complication of peripheral nerve injury, arises from disorganized axonal regeneration and chronic inflammation. Existing treatments provide limited relief. This study aimed to evaluate the therapeutic potential of aligned piezoelectric poly (L-lactide) (PLLA) fibrous scaffolds in preventing neuroma formation by promoting nerve regeneration and mitigating inflammatory and pain-related responses.

**Methods:**

Aligned PLLA fibrous scaffolds were fabricated using electrospinning and characterized for morphology and piezoelectricity. *In vitro*, Schwann cell proliferation, morphology, and expression of myelination-related genes (*Mag*, *Mbp*, *Mpz*) were assessed. *In vivo*, a rat sciatic nerve transection model was used to evaluate autotomy behavior, nerve regeneration, inflammatory and pain-related markers (*TNF-α*, *IL-10*, *SP*, *c-Fos*), and transcriptomic changes.

**Results:**

PLLA scaffolds significantly promoted Schwann cell proliferation and upregulated myelination-related genes *in vitro*. *In vivo*, they reduced autotomy scores and suppressed the expression of inflammatory and nociceptive markers. Histological analyses demonstrated enhanced axonal regeneration and myelination, with greater NF200 and S100 expression, thicker myelin sheaths, and improved structural integrity. Transcriptome analysis revealed upregulation of neuroregenerative genes (e.g., *Mag*, *Mpz*, *Sox10*, *Egr2*) and anti-inflammatory cytokines (e.g., *IL-10*, *TGF-β*), alongside downregulation of proinflammatory and pain-associated genes (e.g., *SP*, *c-Fos*, *Mmp9*, *Tnf-α*).

**Conclusion:**

Aligned piezoelectric PLLA fibrous scaffolds facilitate functional nerve regeneration, promote remyelination, and attenuate neuropathic pain and inflammation. These findings suggest that such scaffolds offer a promising nanomedicine-based strategy for the prevention of traumatic neuroma following peripheral nerve injury.

## 1 Introduction

Traumatic neuroma represents a significant clinical challenge due to its complex pathogenesis and persistent neuropathic pain manifestations (Barnes et al., 2022; Chang et al., 2022; Lin et al., 2023; Santosa et al., 2020). Its development is driven by a complex interplay of mechanisms, including aberrant axonal regeneration, disrupted myelination, chronic inflammatory responses, and so on (Khan et al., 2017; Kong et al., 2022; Richards et al., 2022; Wan et al., 2024). Despite advancements in therapeutic strategies such as pharmacotherapy, nerve blockade procedures, and surgical interventions, current management approaches often exhibit limited effectiveness in providing significant pain relief or preventing the formation of neuromas (Barnes et al., 2022; Kwon et al., 2024; Lin et al., 2023; Odling-Smee, 2023; Senger et al., 2023; Wolvetang et al., 2019). Thus, there is an urgent need to explore novel therapeutic strategies to effectively prevent traumatic neuroma formation.

In recent years, significant attention has been directed toward the development of artificial nerve scaffolds as a preventive measure against neuroma formation. These scaffolds can be engineered to incorporate biophysical cues, such as aligned topography, which has been demonstrated to improve the microenvironment for nerve repair (Casal et al., 2023; Sun et al., 2023; Wang et al., 2024; Xue et al., 2021; Zhao et al., 2020). Notably, electrical stimulation has emerged as a particularly promising biophysical cue for the management of traumatic neuromas. Studies suggest that electrical stimulation can promote myelin formation, mitigate inflammatory responses and alleviate neuropathic pain (Casal et al., 2023; Rajabi et al., 2015; Sun et al., 2023). Conventional electrical stimulation therapies typically use external power sources and supporting wiring systems to ensure the stable delivery of therapeutic electrical cues (Wu et al., 2024), a configuration that has supported its clinical application.

For the prevention of traumatic neuroma, however, a more localized and minimally invasive mode of delivering electrical cues is desirable to match the needs of neural microenvironment regulation after peripheral nerve injury. Recent progress in piezoelectric materials has addressed this demand (Casal et al., 2023; Chen et al., 2022; Li et al., 2015; Pi et al., 2024): these materials can convert endogenous mechanical stimuli (e.g., cell traction forces, physiological activities at the injured site) into localized bioelectric signals, without requiring invasive electrodes or external power connectors. This capability allows piezoelectric materials to retain the therapeutic benefits of electrical stimulation while achieving a more flexible and minimally invasive application mode. Among them, Poly (L-lactide) (PLLA) stands out due to its exceptional piezoelectric properties, biocompatibility, and biodegradability (Zhang et al., 2024), making it a promising material for fabricating scaffolds to prevent traumatic neuroma.

In this study, aligned piezoelectric PLLA fibrous scaffolds were fabricated using electrospinning, a versatile technique known for producing fibrous scaffolds that mimic the nanostructure of the extracellular matrix (ECM). The high surface area and porosity of electrospun nanofibers are particularly advantageous for promoting cell adhesion and growth (Cheng et al., 2025). The effects of these scaffolds on Schwann cell behavior were evaluated *in vitro*. Subsequently, a rat model of sciatic nerve transection was employed to assess the therapeutic efficacy of these scaffolds in inhibiting painful neuroma formation.

## 2 Experimental section

### 2.1 Materials

Poly (L-lactide) (PLLA) and Poly (D, L-lactide) (PDLLA) were purchased from Daigang Biomaterial Co., Ltd., (Jinan, China). Chloroform, rabbit anti-S100 antibody, and mouse anti-NF200 antibody were purchased from Sigma-Aldrich (St. Louis, MO, United States). Cell Counting Kit-8 (CCK-8) and TRIzol were obtained from Beyotime (Shanghai, China). Alexa Fluor 594 anti-rabbit IgG and Alexa Fluor 488 anti-mouse IgG were obtained from Zhongshan Golden Bridge Biotechnology Co. Ltd. (Beijing, China). Reverse transcription kit and SYBR Green Real-time PCR Master Mix were purchased from Takara (Kyoto, Japan) and TOYOBO (Osaka, Japan), respectively. Unless specifically mentioned, the other reagents were purchased from Solarbio (Beijing, China).

### 2.2 Fabrication and characterization of scaffolds

0.5 g of PLLA or PDLLA was dissolved in 10 mL of chloroform and loaded into a syringe pump equipped with a 16-gauge needle. The electrospinning was conducted with the following parameters: voltage set at 15 kV, flow rate maintained at 1.5 mL·h^−1^, roller rotation speed at 2000 rpm, and a working distance of 20 cm. Following the electrospinning process, PLLA and PDLLA fibrous scaffolds, measuring approximately 0.1 mm in thickness, were collected.

The morphology of PLLA and PDLLA scaffolds was imaged by a scanning electron microscope (SEM) (JSM-7900 F, JEOL, Tokyo, Japan). Fiber diameter distribution of the scaffolds was determined from the SEM images using ImageJ software (NIH, Bethesda, MD, United States). The piezoelectric property of PLLA scaffolds was examined using an atomic force microscope (AFM) (Dimension Icon & FastScan Bio, Bruker-Veeco, United States) with a piezoresponse force microscopy (PFM) module.

### 2.3 Schwann cell proliferation assay and morphology observation

Rat Schwann Cells (RSC96) were acquired from the cell bank of the Chinese Academy of Sciences (Shanghai, China) and cultured in DMEM with high glucose, supplemented with 10% fetal bovine serum and 1% penicillin-streptomycin. The electrospun scaffolds were cut into round discs to fit the bottom of standard 24-well tissue culture plates. The scaffolds were sterilized by immersion in 75% ethanol for 30 min followed by UV irradiation for 30 min per side, and subsequently rinsed three times with phosphate-buffered saline (PBS). For cell culture experiments, RSC96 cells were seeded onto three different substrates: tissue culture polystyrene (TCP) as the control group, PDLLA scaffolds, and PLLA scaffolds. Cells were seeded directly onto the surface of the scaffolds or the TCP at a density of 5 × 10^4^ cells/cm^2^. After culturing for 1, 3, and 5 days, the proliferation ability of Schwann cells was evaluated using the CCK-8 assay. Specifically, at each time point, the culture medium was carefully removed and replaced with fresh serum-free medium containing 10% (v/v) CCK-8 reagent. The plates were then incubated at 37 °C in a humidified 5% CO_2_ atmosphere for 2 h. Following incubation, 100 μL of the resulting solution from each well was transferred to a 96-well plate in triplicate. The absorbance was measured at 450 nm using a microplate reader (Bio-Rad, Hercules, CA, United States).

After culturing for 5 days, the morphology of Schwann cells was assessed using immunofluorescent staining. Briefly, the samples were washed with PBS, fixed with 4% paraformaldehyde (PFA), permeabilized with 0.5% Triton X-100, and blocked with 5% BSA. The samples were then incubated overnight at 4 °C with rabbit anti-S100 antibody (1:200) and stained with Alexa Fluor 594 anti-rabbit IgG (1:200) for 2 h at room temperature. The nuclei were stained with 4′,6-diamidino-2-phenylindole (DAPI) at a dilution of 1:1,000.

### 2.4 Quantitative real-time polymerase chain reaction (qRT-PCR)

After culture for 5 days, total RNA was extracted from Schwann cells using TRIzol reagent and subsequently reverse transcribed into cDNA with a reverse transcription kit. Quantitative real-time PCR (qRT-PCR) was conducted using SYBR Green Master Mix on a CFX96™ real-time PCR detection system. The relative gene expression was calculated via the 2^*−*ΔΔCt^ method and normalized by the housekeeping gene glyceraldehyde-3-phosphate dehydrogenase (*GAPDH*). The primers were as follows: myelin associated glycoprotein (*Mag*): forward: 5′- TGC AGT GCC TGT GTG TGG TA-3′, reverse: 5′-CAC AGT CAC GTT GCG GGA AG-3′; myelin basic protein (*Mbp*): forward: 5′-AGA GTC CGA CGA GCT TCA GA-3′, reverse: 5′-CAG GTA CTT GGA TCG CTG TG-3′; myelin protein zero (*Mpz*): forward: 5′-GCT CTT CTC TTC TTT GGT GCT GTC C-3′, reverse: 5′-GGC GTC TGC CGC CCG CGC TTC G-3′; *GAPDH*: forward: 5′-ATG GTG AAG GTC GGT GTG AAC G-3′; reverse: 5′-TTA CTC CTT GGA GGC CAT GTA G-3′.

### 2.5 Animals and surgical procedures

All animal experiments were approved by the Animal Ethics Committee of Peking University People’s Hospital (Approval No. 2020PHE050). Specific-pathogen-free Sprague-Dawley rats (8 weeks old) were obtained from the Beijing Vital River Laboratory Animal Technology Co., Ltd (Beijing, China) and randomly assigned to three groups: PLLA group, PDLLA group, and control (no treatment) group. PLLA and PDLLA scaffolds were shaped into nerve conduits, each measuring 15 mm in length and 2 mm in inner diameter.

All rats were anesthetized using inhalation of 3% isoflurane (RWD Life Science, Shenzhen, China). Following shaving and disinfection, the right sciatic nerve was exposed, and a 15 mm segment was excised at the mid-femur level. In the control group, the proximal nerve stump remained *in situ* without any treatment. In the other groups, the proximal nerve stump was sutured 2 mm into the corresponding nerve conduits. Finally, the muscle and skin incisions were closed.

### 2.6 Autotomy behaviors observation

Autotomy behaviors were assessed by two blinded observers at 2, 4, 6, and 8 weeks postoperatively. Quantitative analysis of autotomy was conducted using the modified Wall Scale. Briefly, a score of 1 was assigned for the removal of two or more nails, with an additional point added for each half toe that was injured. The maximum possible score on this scale was 11, with higher scores indicating more severe injuries.

### 2.7 qRT-PCR, histological evaluation and morphological assessment

All rats were euthanized via carbon dioxide inhalation, and proximal nerve stumps were collected. The expression levels of substance P (*Sp*), c-Fos, and tumor necrosis factor-alpha (*TNF-α*) were measured by qRT-PCR as described above. The primers were as follows: *Sp*: forward: 5′-TGG TCA GAT CTC TCA CAA AGG-3′, reverse: 5′-TGC ATT GCG CTT CTT TCA TA-3′; *c-Fos*: forward: 5′- CAG CCT TTC CTA CTA CCA TTC C-3′, reverse: 5′-ACA GAT CTG CGC AAA AGT CC-3′; *TNF-α*: forward: 5′- ACT GAA CTT CGG GGT GAT TG-3′, reverse: 5′-GCT TGG TGG TTT GCT ACG AC-3′.

For histological evaluation, specimens were fixed in 4% PFA, dehydrated, embedded in paraffin, and cross-cut into 5 μm thick sections. Immunohistochemical staining was conducted using antibodies against Interleukin-10 (*Il-10*) and *TNF-α*, while immunofluorescence staining was performed with antibodies against S100 and NF200. The stained sections were imaged using a slide scanner (Axio Scan Z1, Zeiss, Jena, Germany).

For morphological assessment, specimens were fixed in a 2.5% glutaraldehyde solution, embedded in resin, and cross-cut into 700 nm semithin sections and 70 nm ultrathin sections. The semithin sections were stained with toluidine blue and imaged using a slide scanner (Axio Scan Z1, Zeiss). The ultrathin sections were stained with uranyl acetate and lead citrate and examined using a transmission electron microscope (TEM) (H-800, Hitachi, Tokyo, Japan).

### 2.8 RNA-sequencing

Sequencing library construction, Illumina sequencing, and data analysis were conducted at Novogene Bioinformatics (Beijing, China). Total RNA was extracted from the proximal nerve segments using TRIzol reagent. Following purification, the library was prepared with the NEBNext Ultra RNA Library Prep Kit for Illumina (New England Biolabs, Ipswich, MA, United States). Once the library passed quality control, sequencing was carried out on the Illumina NovaSeq 6000 platform.

### 2.9 Statistical analysis

All numerical data are presented as the mean ± standard deviation (SD). Statistical analysis was conducted using GraphPad Prism 9 software (GraphPad Software, Inc., La Jolla, CA, United States). Differences between multiple groups were assessed using one-way analysis of variance followed by Tukey’s *post hoc* tests. Results were considered statistically significant at *P* < 0.05.

## 3 Results and discussion

### 3.1 Characterization of scaffolds

**FIGURE 1:**
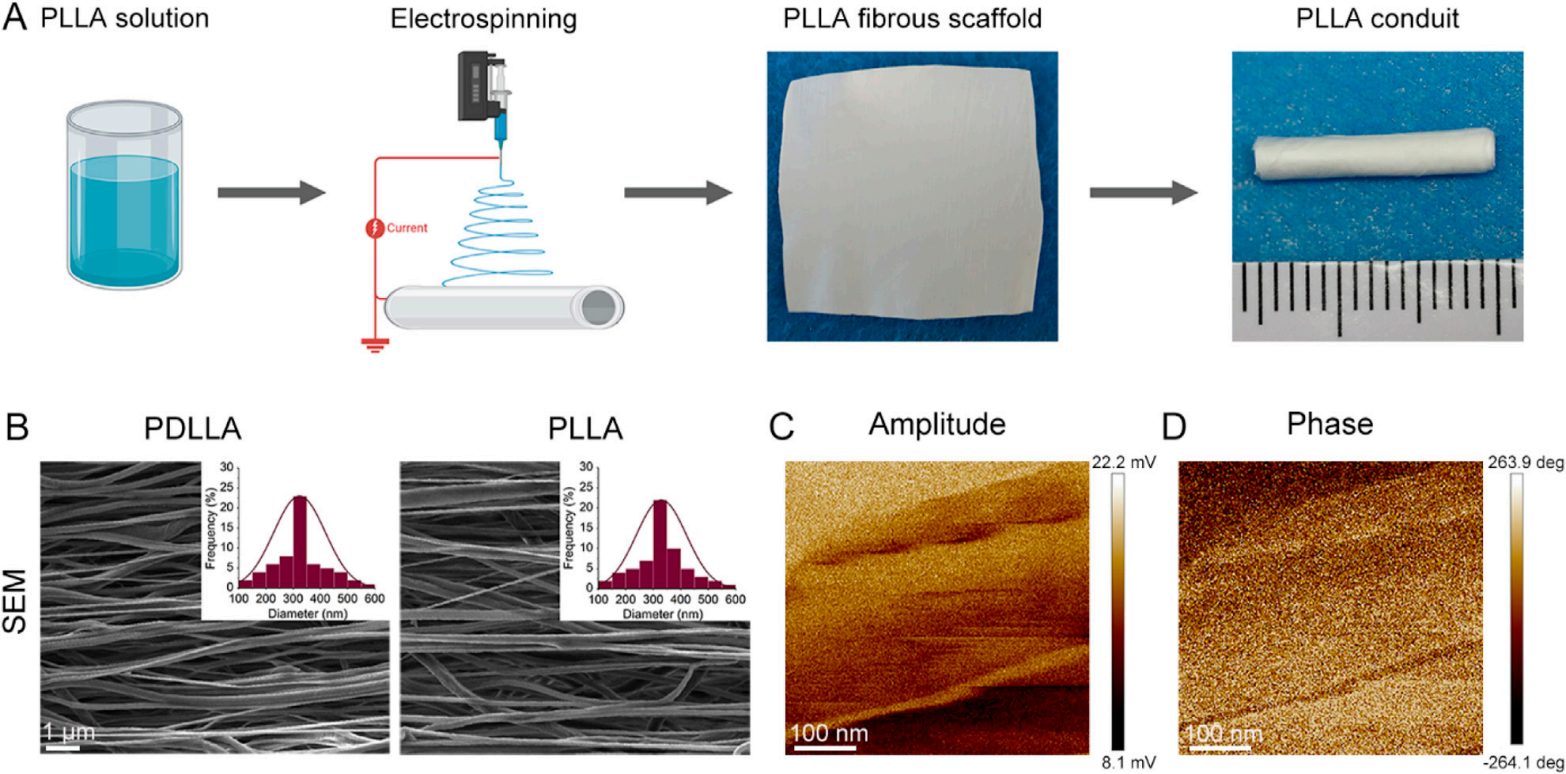
Scaffolds characterization. **(A)** Scheme of the preparation process of PLLA fibrous scaffolds and PLLA nerve conduit. **(B)** SEM image and fiber diameter distribution of PDLLA and PLLA fibrous scaffolds. **(C)** PFM amplitude image and **(D)** phase image of PLLA fibrous scaffolds.

The optical images of PLLA fibrous scaffolds and PLLA nerve conduit are presented in Figure 1A. As shown in the SEM images (Figure 1B), the fibers in both PDLLA and PLLA fibrous scaffolds are arranged in an oriented manner. And the fiber diameters of PDLLA and PLLA exhibit similar distribution profiles, with most fibers measuring approximately 300–400 nm. Additionally, PFM measurements confirmed the piezoelectric activity in PLLA fibrous scaffolds (Figures 1C,D).

### 3.2 Proliferation, morphology and myelination of Schwann cells on PLLA fibrous scaffolds

**FIGURE 2:**
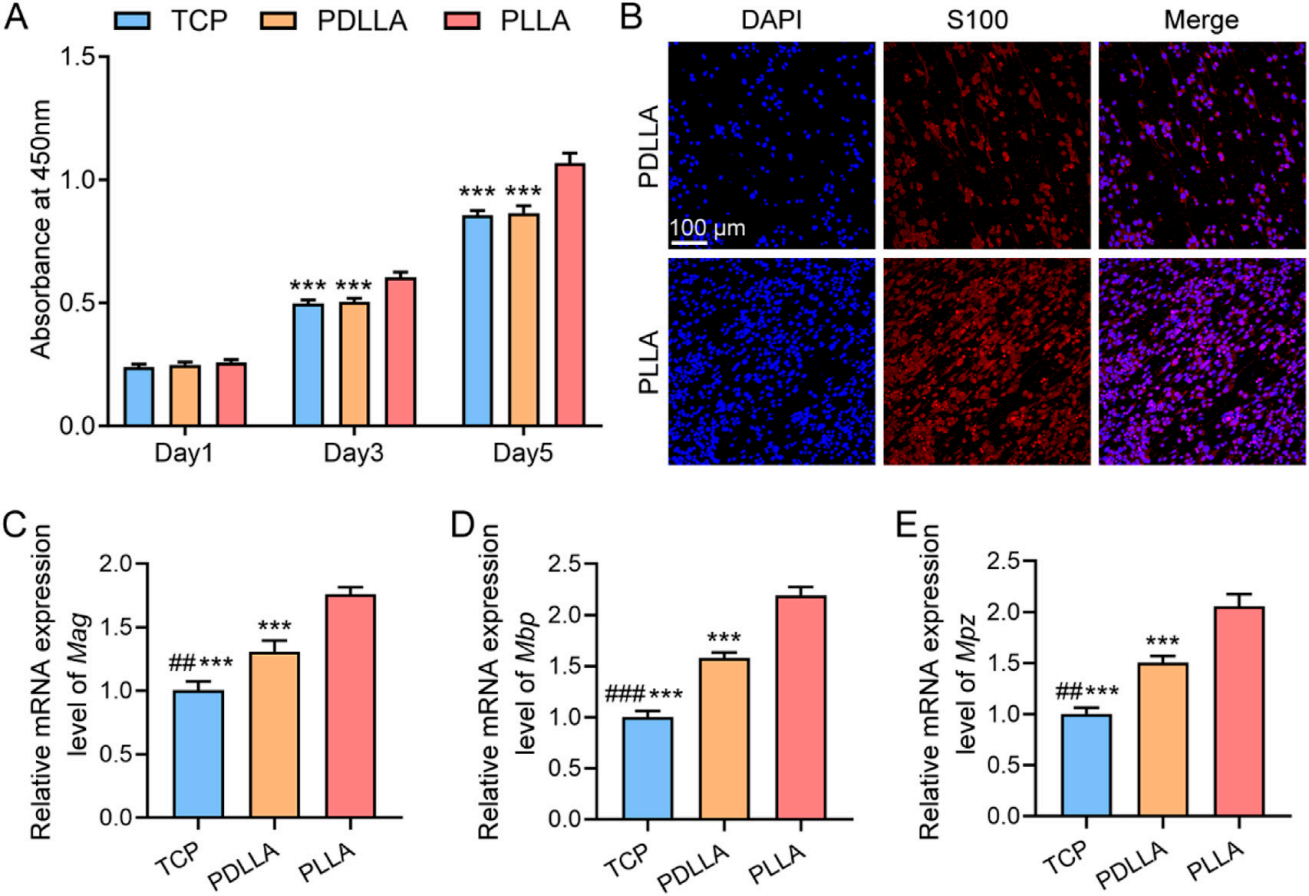
Effect of PLLA fibrous scaffolds on the behaviors of Schwann cells. **(A)** CCK-8 assay results of Schwann cells on days 1, 3, and 5. **(B)** Immunofluorescence images of Schwann cells on day 5. Cells stained with S100 (red), and nuclei stained by DAPI (blue). Relative mRNA expression levels of **(C)** *Mag*, **(D)** *Mbp*, and **(E)** *Mpz* on day 5. (*n* = 3, ***P < 0.001, vs. PLLA group; ##*P* < 0.01, ###*P* < 0.001, vs. PDLLA group).

Schwann cells, the primary glial cells responsible for myelination in the peripheral nervous system (PNS), were selected as the cellular model to evaluate the cytocompatibility and pro-myelination potential of our prepared scaffolds (Li et al., 2015). The CCK-8 assay results (Figure 2A) revealed that Schwann cells exhibited continuous proliferation in TCP, PDLLA, and PLLA groups. After 1 day of culture, no significant differences in proliferative capacity were observed among the three groups (all *P* > 0.05). After 3 and 5 days of culture, the PLLA group demonstrated significantly enhanced Schwann cell proliferation compared to the PDLLA and control groups (all *P* < 0.001). And no significant difference was found between the PDLLA and TCP groups (all *P* > 0.05). Morphological analysis (Figure 2B) revealed that Schwann cells exhibited spindle-shaped morphologies aligned with the fiber orientation. Additionally, the cell density in the PLLA group was higher than that in the PDLLA group. This observation was consistent with the results of CCK-8 assay (Figure 2A). *Mag* serves as an early myelination biomarker, while *Mbp* and *Mpz* are the primary structural proteins of myelin. To assess the impact of PLLA scaffolds on myelination, we measured the gene expression levels of *Mag*, *Mbp*, and *Mpz* using qRT-PCR. As shown in Figures 2C–E, the relative mRNA expression levels of *Mag*, *Mbp*, and *Mpz* were significantly upregulated in the PLLA group compared to the PDLLA and TCP groups (all *P* < 0.001). Additionally, significant differences were observed between the PDLLA and TCP groups (*P* < 0.01 or *P* < 0.001). These results collectively demonstrate that PLLA scaffolds are more conducive to Schwann cell proliferation and myelination.

### 3.3 Alleviation of autotomy behaviors, pain-related markers expression, and inflammatory reactions with PLLA fibrous scaffolds

**FIGURE 3:**
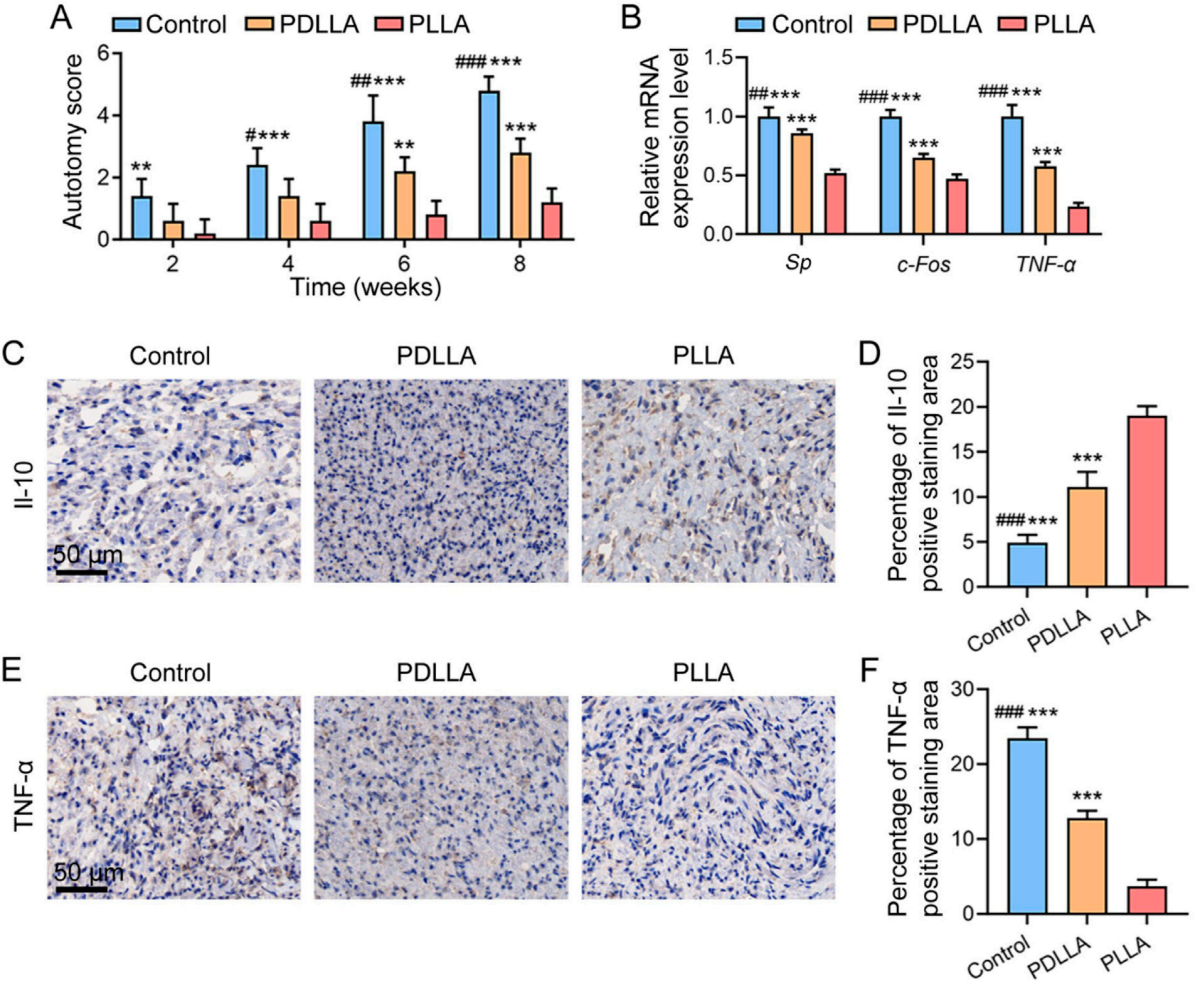
Effects of PLLA fibrous scaffolds on autotomy behaviors, pain-related marker expression, and inflammatory responses. **(A)** Autotomy scores assessed at 2-, 4-, 6-, and 8-weeks post-operation. **(B)** Relative mRNA expression levels of *Sp*, *c-Fos*, and *TNF-α* at 4 weeks post-operation. Immunohistochemical staining images of **(C)** *Il-10* and **(D)** *TNF-α* at 8 weeks post-operation. Statistical analysis of **(E)** the percentage of *Il-10*-positive staining area and **(F)** the percentage of *TNF-α*-positive staining area. (*n* = 5, **P < 0.01, ***P < 0.001, vs. PLLA group; #*P* < 0.05, ##*P* < 0.01, ###*P* < 0.001, vs. PDLLA group).

The autotomy score is widely used to quantify neuropathic pain severity following peripheral nerve injury (Yon et al., 2023). As shown in Figure 3A, at 2 weeks post-operation, the PLLA group demonstrated significantly lower scores compared to the Control group (*P* < 0.01). By 4 weeks, the PLLA group continued to show significantly lower scores than the Control group (*P* < 0.001), while the PDLLA group also began to exhibit significantly reduced scores in comparison to the Control group (*P* < 0.05). At 6 and 8 weeks, the PLLA group displayed significantly lower scores than both the PDLLA and Control groups (*P* < 0.01 or *P* < 0.001), with the PDLLA group also having significantly reduced autotomy scores compared to the Control group (*P* < 0.01 or *P* < 0.001).

Given that autotomy behavior serves as an indirect measure of pain, we complemented these observations with more precise molecular markers to strengthen our findings. Specifically, we selected *Sp* and *c-Fos* as pain-related biomarkers, as prior studies have shown their expression decreases when neuropathic pain is alleviated (Finnerup et al., 2021; He et al., 2021; Sakai et al., 2005). Additionally, we included TNF-α in our analysis due to its well-established involvement in peripheral and central sensitization across diverse neuropathic pain models (Choi et al., 2023; Khan et al., 2017; Shelton et al., 2005). At 4 weeks post-operation, we assessed the relative mRNA expression levels of *SP*, *c-Fos*, and *TNF-α* in the proximal nerve stump. As illustrated in Figure 2B, the PLLA group exhibited significantly lower expression levels of these biomarkers compared to both the PDLLA and control groups (all *P* < 0.001). In addition, notable differences were observed between the PDLLA and control groups (*P* < 0.01 or *P* < 0.001).

Inflammation in the PNS is known to play a critical role in the onset and persistence of neuropathic pain (Khan et al., 2017; Zelenka et al., 2005). *Il-10*, a potent anti-inflammatory cytokine, has a broad spectrum of anti-inflammatory effects and is implicated in neuropathic pain by suppressing nuclear factor kappa B (*NF-κB*) activity and reducing the synthesis of pro-inflammatory cytokines, such as *Il-1β* and *TNF-α* (Khan et al., 2017; Zelenka et al., 2005). Consequently, *Il-10* has been explored as a potential therapeutic agent for neuropathic pain. At 8 weeks post-operation, we evaluated the expression of *Il-10* and *TNF-α* in the proximal nerve stump using immunohistochemical staining (Figures 3C,E). Quantitative analysis revealed that the percentage of *Il-10* positive staining area in the PLLA group was significantly higher than in both the PDLLA and control groups (all *P* < 0.001) (Figure 3D). Similarly, the PDLLA group showed a significantly greater *Il-10* positive staining area compared to the control group (P < 0.001) (Figure 3D). Conversely, the percentage of *TNF-α* positive staining area in the PLLA group was significantly lower than in both the PDLLA and control groups (all *P* < 0.001), with the PDLLA group also exhibiting a significantly lower *TNF-α* positive staining area compared to the control group (P < 0.001) (Figure 3F). These findings underscore the potential therapeutic benefits of PLLA fibrous scaffolds in modulating inflammatory responses and alleviating neuropathic pain.

### 3.4 Nerve regeneration with PLLA fibrous scaffolds

**FIGURE 4:**
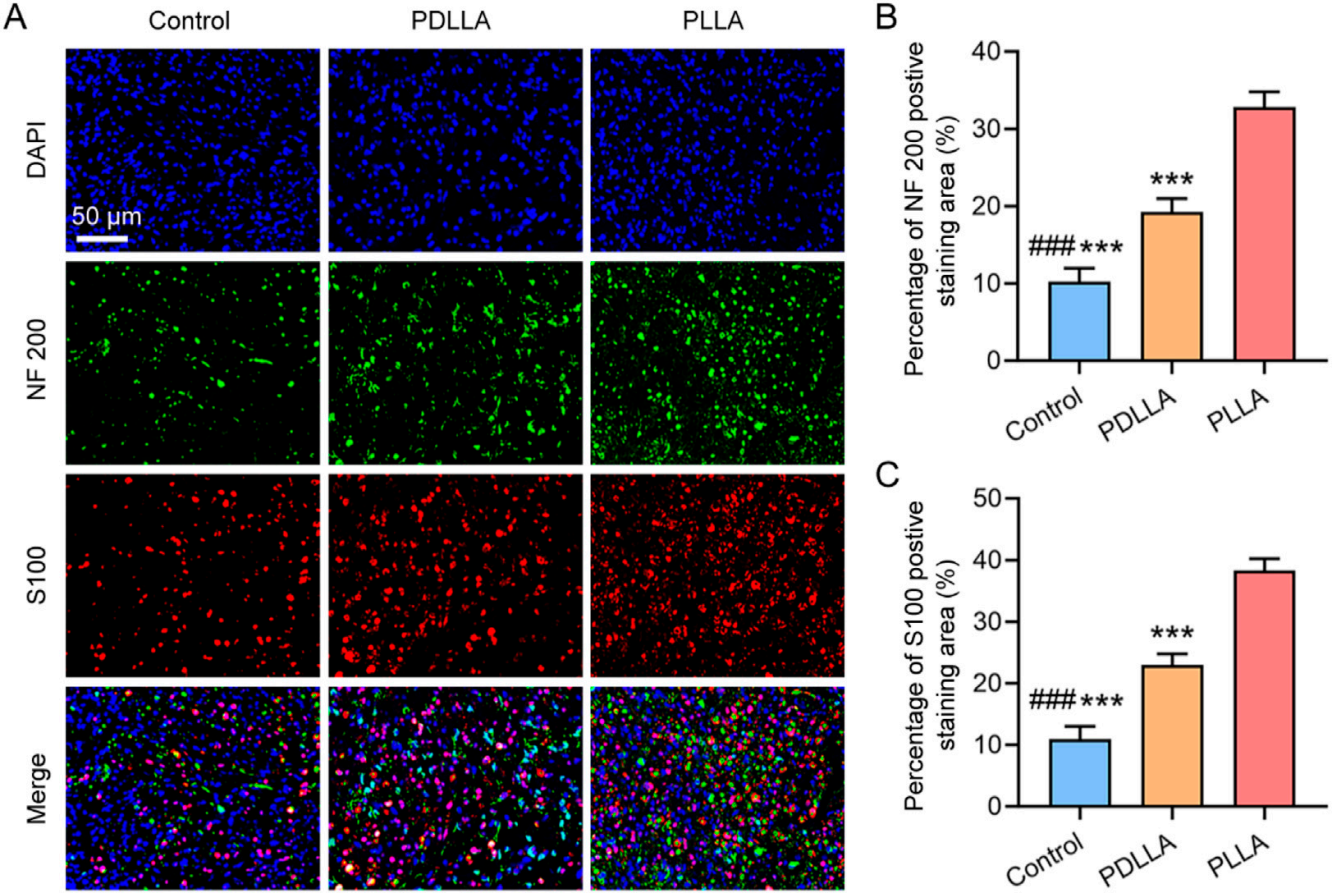
Immunofluorescence analysis of the proximal nerve stump at 8 weeks post-operation. **(A)** Double immunofluorescent staining showing the axon marker NF200 (green) and the Schwann cell marker S100 (red). Statistical results of **(B)** the percentage of NF200 positive staining area and **(C)** the percentage of S100 positive staining area. (*n* = 5, ***P < 0.001, vs. PLLA group; ###*P* < 0.001, vs. PDLLA group).

To evaluate the histological characteristics of nerve regeneration under different conditions (Liu et al., 2022; Wang et al., 2024), we harvested the proximal nerve stumps at 8 weeks post-operation and performed immunofluorescent staining for NF200 and S100 to detect axons and Schwann cells, respectively (Figure 4A). Quantitative analysis revealed that the percentage of NF200 positive staining area in the PLLA group was significantly higher than that in both the PDLLA and control groups (all *P* < 0.001), while the PDLLA group also showed a significantly greater NF200 positive staining area compared to the control group (*P* < 0.001) (Figure 4B). Similarly, the S100 positive staining area in the PLLA group was significantly larger than that in the PDLLA and control groups (all *P* < 0.001), with the PDLLA group also exhibiting a higher S100 positive staining area than the control group (*P* < 0.001) (Figure 4C). These findings indicate that the PLLA scaffold supported the formation of a structured neural tissue within the conduit. The higher density of NF200-positive axons and S100-positive Schwann cells, compared to the disorganized neuroma-like tissue in the control group, suggests that the scaffold provided a guided and contained environment for neural ingrowth. This organized architecture is a key histological indicator of successful neuroma prevention, as it contrasts sharply with the chaotic proliferation that characterizes painful neuromas.

### 3.5 Axonal myelination with PLLA fibrous scaffolds

**FIGURE 5:**
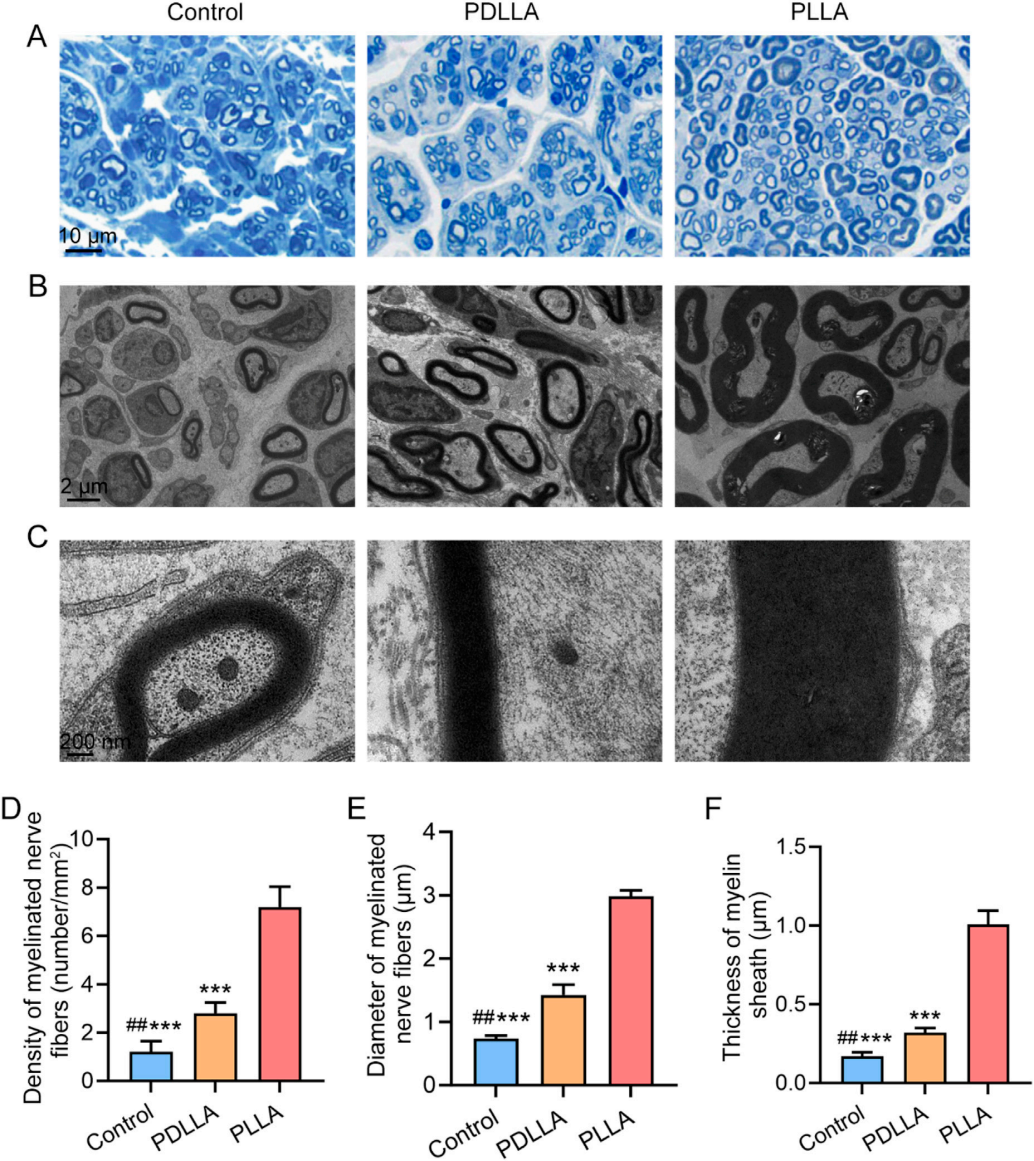
Morphological evaluation of the proximal nerve stump at 8 weeks post-operation. **(A)** Toluidine blue staining images. **(B)** TEM images. **(C)** Zoom-in micrographs of the images shown in **(B)**. Statistical results of **(D)** the density of myelinated nerve fibers, **(E)** the diameter of myelinated nerve fibers and **(F)** the thickness of myelin sheath. (*n* = 5, ***P < 0.001, vs. PLLA group; ##*P* < 0.01, vs. PDLLA group).

The impact of PLLA fibrous scaffolds on axonal myelination was further investigated. The myelin sheath, a spiral structure enveloping axon, serves as an essential insulating layer (Chen et al., 2015; Tang et al., 2024). This protective barrier not only shields axons from mechanical injury but also prevents aberrant electrical signaling between neighboring nerves, thereby reducing the risk of abnormal nerve growth and neuroma formation. To evaluate axonal myelination, toluidine blue staining was performed to assess the density of myelinated nerve fibers (Figure 5A). The PLLA group demonstrated a significantly higher density of myelinated nerve fibers compared to both the PDLLA and Control groups (all *P* < 0.001), with a notable difference also observed between the PDLLA and Control groups (*P* < 0.01) (Figure 5D). To further analyze the ultrastructure of the myelin sheath, TEM was utilized. As depicted in Figures 5B,C, the myelin lamellae in the PLLA group exhibited a dense and compact arrangement. Quantitative analysis revealed that the diameter of myelinated nerve fibers in the PLLA group was significantly larger than that in the PDLLA and Control groups (all *P* < 0.001), with the PDLLA group also showing a significantly greater diameter compared to the control group (*P* < 0.01) (Figure 5E). Additionally, the thickness of the myelin sheath, a key marker of axonal maturity, was significantly greater in the PLLA group than in the other conditions (all *P* < 0.001), and the PDLLA group also displayed a significantly thicker myelin sheath compared to the Control group (*P* < 0.01) (Figure 5F). These findings collectively indicate that the PLLA scaffold facilitated the formation of a well-organized neural structure. The presence of densely packed and uniformly myelinated fibers within the conduit, as opposed to the haphazard and variably myelinated fiber bundles found in neuromas, demonstrates that the scaffold promoted a structured regenerative outcome. This organized myelination supports the functional conclusion that the scaffold mitigates the risk of painful neuroma development by preventing the disorganized axonal sprouting that defines the condition.

### 3.6 Mechanisms of PLLA fibrous scaffolds in inhibiting traumatic neuroma formation

**FIGURE 6:**
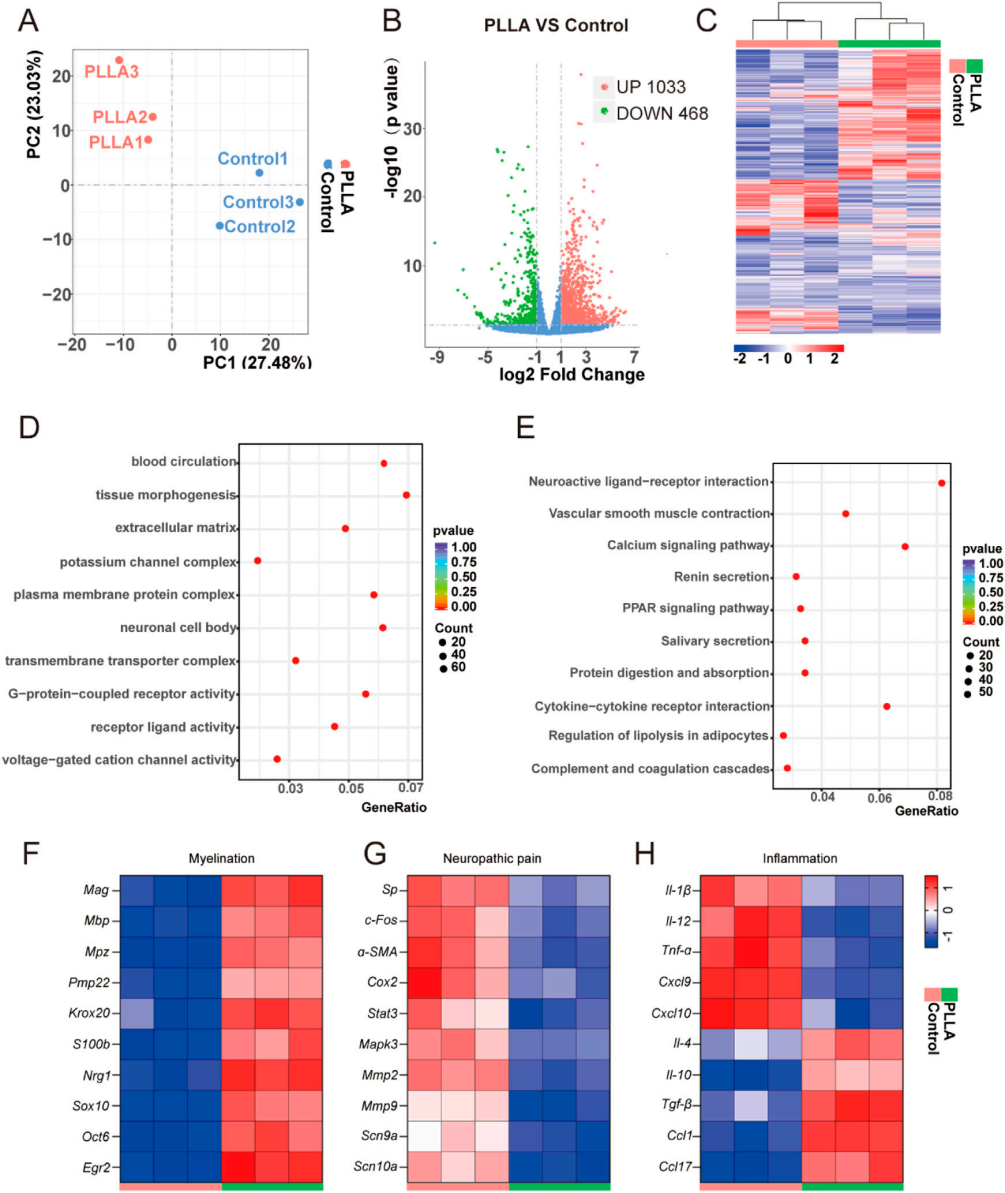
RNA-sequencing analysis of proximal nerve stumps treated with PLLA fibrous scaffolds. **(A)** Principal component analysis (PCA) of transcriptomic profiles from control and PLLA groups. **(B)** Volcano plots showing differentially expressed genes (DEGs), with upregulated genes in red and downregulated genes in blue. **(C)** Hierarchical clustering analysis of gene expression patterns between control and PLLA groups. **(D,E)** Gene Ontology (GO) and Kyoto Encyclopedia of Genes and Genomes (KEGG) enrichment analyses of DEGs. Heatmaps illustrating differential expression of genes associated with **(F)** myelination, **(G)** neuropathic pain, and **(H)** inflammation, including proinflammatory and anti-inflammatory cytokines.

To investigate the mechanisms underlying the therapeutic effects of PLLA fibrous scaffolds, we performed RNA-sequencing on proximal nerve stumps at 8 weeks post-operation. Principal component analysis (PCA) revealed distinct transcriptomic profiles between the PLLA and control groups (Figure 6A). Volcano plots identified 1,501 differentially expressed genes (DEGs) following PLLA scaffold treatment, comprising 1,033 upregulated and 468 downregulated genes (Figure 6B). Hierarchical cluster analysis highlighted gene expression differences between the control and PLLA groups (Figure 6C). Gene ontology (GO) and Kyoto Encyclopedia of Genes and Genomes (KEGG) enrichment analyses revealed that PLLA scaffold treatment modulated biological processes critical for neuropathic pain relief and neural maturation (Figures 6D,E). Specifically, genes associated with myelination, including *Mag*, *Mbp*, *Mpz*, *Pmp22*, *Krox20*, *S100b*, *Nrg1*, *Sox10*, *Oct6*, and *Egr2*, showed higher expression in the PLLA group compared to the control group (Figure 6F). Conversely, genes linked to neuropathic pain, such as *Sp*, *c-Fos*, *α-SMA*, *Cox-2*, *Stat3*, *Mapk3*, *Mmp2*, *Mmp9*, *Sxn9a*, and *Scn10a*, were downregulated (Figure 6G). Additionally, the expression of proinflammatory cytokines, including *Il-1β*, *Il-12*, *Tnf-α*, *Cxcl-9*, and *Cxcl-10*, was reduced in the PLLA group (Figure 6H). In contrast, anti-inflammatory cytokines such as *Il-4*, *Il-10*, *Tgf-β*, *Ccl-1*, and *Ccl-17* were upregulated in the PLLA group (Figure 6H). The modulation of this inflammatory milieu is critically linked to the inhibition of fibrosis, a key barrier to successful neural integration (Sarhane et al., 2019). Pro-inflammatory cytokines such as TNF-α and IL-1β are potent activators of fibroblasts and drivers of collagen deposition. Their downregulation, coupled with the upregulation of anti-fibrotic cytokines like IL-10 and TGF-β, indicates that the PLLA scaffold creates an environment that is hostile to the formation of dense fibrotic scar tissue. This reduction in fibrosis is instrumental in preventing the entrapment of regenerating neural elements, a hallmark of painful neuromas. Collectively, our results demonstrate a multifaceted mechanism of action for the PLLA scaffolds. However, it is important to note that traumatic neuroma formation is a complex process involving multiple cell types and molecular pathways, and this study may not have addressed all contributing factors. Additionally, while this study utilized a rat sciatic nerve transection model, further validation across diverse animal models would improve the generalizability of the findings.

## 4 Conclusion

Herein, aligned piezoelectric PLLA fibrous scaffolds were successfully fabricated through electrospinning technology, and their potential in inhibiting traumatic neuroma formation was evaluated using *in vitro* and *in vivo* models. *In vitro* studies demonstrated that these scaffolds significantly enhanced Schwann cell proliferation and upregulated the expression of myelination-related genes, including *Mag*, *Mbp*, and *Mpz*. *In vivo* experiments revealed these scaffolds synergistically inhibits the formation of traumatic neuroma after sciatic nerve transection and effectively alleviates neuropathic pain. Taken together, these findings establish aligned piezoelectric PLLA fibrous scaffolds as a promising biomaterial platform for the prevention of traumatic neuroma formation.

## Data Availability

The raw data supporting the conclusions of this article will be made available by the authors, without undue reservation.
